# Development of single-cell ICP-TOFMS to measure nanoplastics association with human cells†

**DOI:** 10.1039/d3en00681f

**Published:** 2023-11-09

**Authors:** Lyndsey Hendriks, Vera M. Kissling, Tina Buerki-Thurnherr, Denise M. Mitrano

**Affiliations:** a TOFWERK AG Schorenstrasse 39 3645 Thun Switzerland; b Laboratory for Particles Biology Interactions, Swiss Federal Laboratories for Materials Science and Technology (Empa) Lerchenfeldstrasse 5 CH-9014 St. Gallen Switzerland; c Department of Environmental Systems Science, ETH Zurich Universitätstrasse 16 8092 Zurich Switzerland denise.mitrano@usys.ethz.ch

## Abstract

Nanoplastics, solid polymer particles smaller than 1 μm, are suspected to be widely present in the environment, food and air, and may pose a potential threat to human health. Detecting nanoplastics in and associated with individual cells is crucial to understand their mechanisms of toxicity and potential harm. In this context, we developed a single-cell inductively coupled plasma time-of-flight mass spectrometry (sc-ICP-TOFMS) method for the sensitive and rapid quantification of metal-doped model nanoplastics in human cells. By providing multi-elemental fingerprints of both the nanoplastics and the cells, this approach can be advantageous in laboratory toxicological studies as it allows for the simultaneous acquisition of a full mass spectrum with high time resolution. As a proof-of-concept study, we exposed two different human cell lines relevant to inhalation exposures (A549 alveolar epithelial cells and THP-1 monocytes) to Pd-doped nanoplastics. The sc-ICP-TOFMS analysis revealed a similar dose-dependent endocytotic capacity of THP-1 and A549 cells for nanoplastics uptake, and particle internalization was confirmed by transmission electron microscopy. Moreover, single-cell quantification showed that a considerable proportion of the exposed cells (72% of THP-1; 67% of A549) did not associate with any nanoplastics after exposure to 50 μg L^−1^ for 24 h. This highlights the importance to include single-cell analysis in the future safety assessment of nanoplastics in order to account for heterogeneous uptake within cell populations and to identify the origins and response trajectories of nanoplastics in biological tissues. In this regard, sc-ICP-TOFMS can be a powerful approach to provide quantitative data on nanoplastics–cell associations at single cell level for a large number of individual cells.

## Abstract 2

Nanoplastics, solid polymer particles smaller than 1 μm, are suspected to be widely present in the environment, food and air, and may pose a potential threat to human health.

## Graphical abstract

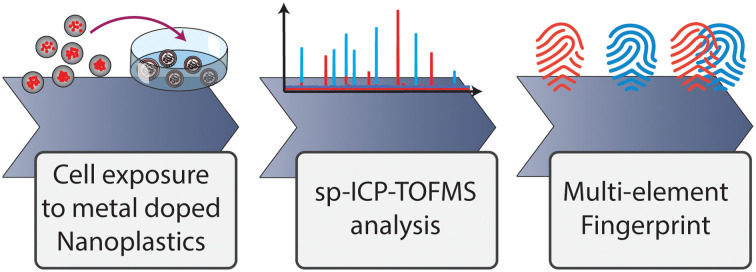

**Environmental significance**

Nanoplastics are suspected to be widely present in the environment, food and air, and may pose a potential threat to human health. Detecting nanoplastics in and associated with individual cells is crucial to understanding their mechanisms of toxicity and potential harm. In this context, we developed a single-cell inductively coupled plasma time-of-flight mass spectrometry (sc-ICP-TOFMS) method for the sensitive and rapid measurement of model nanoplastics associated with cells. Understanding the association of nanoplastics with cells, especially on a cell-by-cell basis, is a powerful approach to assess the heterogeneity of nanoplastics association in biological systems. This is an initial step to appreciating the diversity of nanoplastics–cell interactions in a high-throughput fashion, which can be applied not only to human cell lines, as done here, but also to other cells and biological systems.

## 1. Introduction

The physical and chemical characteristics of the different size classes of plastic pollution (macroplastics, microplastics and nanoplastics) will result in diverse fate and hazards.^1^ When plastic particles become smaller, there are a number of different processes which influence their environmental risks, including interactions with and adverse effects to organisms. There is little evidence that micrometer-sized particles can cross biological barriers, such as the lung or the gut lining, in large amounts and therefore these particles are anticipated to largely be excreted from the body.^2^ Nevertheless, some microplastics have been observed in human tissues.^3,4^ However, below 400 nm, the biological fate of particles changes. Particle uptake into cells is possible,^5^ biological barriers can be crossed and systemic uptake of particles can occur,^6–8^ which has also been shown for nanoplastics.^9–11^ Many studies on the uptake and effects of several polymeric nanomaterials using different cell types have been performed to show that their uptake into cells is comparable to other nanomaterials.^12–16^ Both particle size and surface chemistry (including *e.g.*, eco- or protein corona formation^17^) play crucial roles in particle translocation and uptake.^16^ Moreover, the diversity of cells can drive divergent particle uptake mechanisms, as demonstrated for 40 nm carboxylated polystyrene particles that showed distinct entry routes in J774A.1 macrophages (macropinocytosis, phagocytosis, and clathrin-mediated endocytosis) and A549 alveolar epithelial cells (caveolin- and clathrin-mediated endocytosis).^18^ In general, different uptake mechanisms have been described in a variety of cells but since most studies used primary polystyrene nanoplastics, further work is needed to understand potential differences in uptake pathways due to different polymer types or secondary nanoplastics.^19^

Investigating biological interactions between particles and cells is not a new field of research, and thus some mechanistic understanding of how nanoplastics may associate with cells can be gleaned from fields such as nanomedicine and nanotoxicology of engineered nanomaterials.^20,21^ Nanosafety in both these domains focuses on developing methods to understand nanomaterial–cell interactions better. Researchers are also working on techniques for characterizing particles in complex mixtures, as well as in organisms and cellular models. Furthermore, they aim to investigate the chemical and biological effects resulting from the interplay of physicochemical properties of nanomaterials during exposure. These effects include biodistribution, biotransformation, accumulation, and toxicity. This underpins the idea that some of the concepts and approaches used to evaluate other nano-sized objects will also be applicable for nanoplastics.

Assessing nanoplastics association with cells is more analytically challenging than other metal-based nanomaterials because of their chemical composition. Plastics particles down to the μm-size can be detected by monitoring the carbon content by ICP-MS analysis, however due to the low sensitivity of ICP-MS for carbon, this approach is not sufficient to target nano-sized plastics.^22–24^ Labelled or doped nanoplastics have been previously used as an approach to trace and characterize nanoplastics in complex media and biological tissues, including the use of fluorescent dyes, isotope labels^25^ and trace metals.^26^ Fluorescence labeling is a common method due to ease of detection by simple optical methods, but limitations include potential modification of particle properties and bio-interactions from incorporated dyes, stability of fluorescence, leakage of dyes from the particle as well as limited sensitivity to detect and quantify individual or small particles.^27–29^ Using metal-doped nanoplastics has been shown to have several key advantages over the other options, since existing standard methods for trace metals analysis exist and can be exploited for measuring metal-doped plastics in a variety of technical and environmental systems, including biological tissues and cells.^9,10,30–36^ In these instances, a sample is typically measured by digesting the sample matrix by microwave-induced acid digestion and the metal content analyzed by ICP-MS to back-calculate plastic concentrations in a given sample. In terms of assessing particle fate and transport, or particle uptake and depuration in organisms, these metrics are often sufficient for the study at hand to assess behavior of nanoplastics in the target system. However, this bulk analysis does not allow one to measure uptake on a cell-by-cell basis, or to assess the number of nanoplastics associated with an individual cell.

By further discretizing the information obtained by ICP-MS on undigested samples, such as with single-particle ICP-MS (sp-ICP-MS) or single-cell ICP-MS (sc-ICP-MS), one is able to assess individual nanoparticles or nanoparticles associated with individual cells.^37,38^ In both cases, the goal is to analyze the sample on a single entity basis, but the difference between single-cell or single-particle ICP-MS lies in the sample being analyzed and, in some cases, a change in the sample introduction system. After nebulization of the sample, the individual entities (nanoparticles and/or cells) are carried in droplets into the plasma, where they are subsequently vaporized, atomized and finally ionized. Each individual entity generates an ion cloud, which is recorded as a spike above the background. The frequency of the detected spikes is proportional to the number concentration of the single entities, while the magnitude of the spike correlates to the mass of the single entity.^39,40^ Often, a quadrupole ICP-MS is used and thus only one (or, with fast single-particle ICP-MS, two)^41^ elements can be recorded for each entity event. Alternatively, by utilizing an ICP time-of-flight mass spectrometer (ICP-TOFMS), the entire mass spectrum can be obtained simultaneously for each single entity event, allowing one to determine the entire elemental fingerprint of individual particles, cells, or aggregates.^42–48^ In this way, association of metal-doped nanoplastics can be measured with individual cells, providing much better resolution for particle association with cells than with other methods.^49^

It should be noted that the groundwork for sc-ICP-TOFMS was laid over a decade ago by Bandura *et al.* who introduced the concept of time-resolved analysis of individual cells, thereby paving the way for mass cytometry.^50^ Mass cytometry instruments, also known as CyTOF, are ICP-MS instruments with a time-of-flight detector specifically designed for single-cell analysis. Because the technique falls short in detecting the majority of the intrinsic elements present in single cells (analytes with masses <75 amu), metal-isotope labels are used to tag cells, enabling their detection based on the associated metal labels. Nevertheless, technological advancements of this approach have revolutionized the field, enabling high-throughput analysis of single cells in the micrometer range. Consequently, mass cytometry has now found applications in large-scale multicenter clinical studies.^51^ However, when specifically exploring the metallome,^52^ which refers to the complete inventory of metal species present in a biological system encompassing essential metals, trace metals, and metalloids, and their roles in various biological processes, the limitations of mass cytometry to detect the lower mass elements becomes apparent. Thus, in order to measure the majority of all endogenous cellular elements, an ICP-TOFMS enabling the acquisition of a full mass spectrum ranging from *m*/*z* = 7 to 280 is advantageous.

In this manuscript, we developed a sc-ICP-TOFMS method to assess nanoplastics association with human cells grown in culture both to show a proof of principle of the technique and to compare association/uptake of nanoplastics across two of the most relevant cell types (*i.e.* monocytes/macrophages and epithelial cells) for uptake and interaction with nanoparticles at biological barriers. The focus of this study was set on lung cells since inhalation is one of the main exposure routes for nanoplastics uptake besides ingestion and dermal contact and the lung is particularly sensitive to nanoparticle exposure.^53^ Moreover, monocytes/macrophages were studied as they are the first line of defense of the innate immune system and immediately react with nanoparticles, triggering a variety of immune responses.^54^ Metal-doped nanoplastics allowed us to harness the specificity of metal analytics in the context of plastics research, and by using an ICP-TOFMS we could confidently pair nanoplastics events with cell events. More specifically, we aimed to 1) optimize the sample preparation and sample introduction systems for high particle and cell recovery, 2) show the utility of sc-ICP-TOFMS to measure individual nanoplastics with individual cells and 3) understand dose-dependent association of model nanoplastics with human monocytes (THP-1) and alveolar epithelial cells (A549). Collectively, this work makes a strong foundation for a powerful analytical method to explore nanoplastics exposures at the single cell level.

## 2. Materials and methods

### 2.1 Model nanoplastics synthesis and characterization

Palladium (Pd) doped nanoplastics were synthesized in-house as previously published by Mitrano *et al.*^26^ In brief, nanoplastics were created through emulsion polymerization where a Pd-containing salt was introduced together with an initiator into a reactor containing acrylonitrile and SDS to form nanoplastics with a metal content of approximately 0.3 wt% Pd. Subsequently, a shell of polystyrene was grown on top of this core particle to achieve a final diameter of approximately 200 nm. Pd-doped nanoplastics were characterized prior to use in the exposure studies in terms of particle size, surface charge and metal content. Particle hydrodynamic diameter and stability was determined by Dynamic Light Scattering (DLS) and zeta potential, respectively (Malvern Zetasizer). Previous studies have tested the stability of the Pd-tracer incorporated into the nanoplastics to ensure no leaching of metal occurred over the duration of the exposure experiments.^55^

### 2.2 Cell culture preparation and nanoplastics exposure

The human alveolar epithelial cell line A549 (CCL-185, ATCC) and the human acute monocytic leukemia cell line THP-1 (TIP-202, ATCC) were used for nanoplastics–cell association studies. Cells were maintained in cell culture medium (CM), which was RPMI-1640 medium (Sigma-Aldrich, R0883) supplemented with 10% fetal bovine serum (Sigma-Aldrich F9665), 0.2 mg mL^−1^l-glutamine (Sigma G7513) and 1% penicillin–streptomycin (Sigma, P4458). The particle stock solutions were diluted with CM to the required concentrations immediately prior to the cell treatments. For exposure studies, cells were first seeded (10 × 10^6^ A549 cells in 10 mL CM or 8 × 10^6^ THP-1 cells in 8 mL per T75 flask) and cultivated for 4 h before addition of the same volume of CM containing double-concentrated particles (*i.e.* 0, 1, 10 and 100 μg L^−1^ nanoplastics) to achieve final concentrations of 0, 0.5, 5 and 50 μg L^−1^ nanoplastics. This approach was used to achieve the same treatment procedure for both cell types since a simple medium exchange was not possible for non-adherent THP-1 cells. Three biologically independent replicates were performed for all cell exposures. After 24 h of exposure, THP-1 suspension cells were directly collected while adherent A549 cells were first washed three times with phosphate buffered saline (PBS) to remove non-associated particles and then trypsinized. In order to remove unbound nanoplastics, cells were washed twice with PBS, fixed with 4% paraformaldehyde (ROTI-Histofix 4%, P087.5) for 10 min and washed again twice with ultrapure water. Cells were shipped and stored at 4 °C until sc-ICP-TOFMS analysis. Prior to sc-ICP-TOFMS analysis, the cells were again washed with MilliQ water (4×) and diluted by a factor of 10 in MilliQ water to achieve appropriate particle concentrations for analysis (see Fig. S1 in the ESI†).

### 2.3 ICP-TOFMS procedure in single-cell mode

An ICP-TOFMS instrument (icpTOF S2, TOFWERK AG, Thun, Switzerland) was used for all measurements and has already been described elsewhere.^23,56^ The icpTOF S2 ran at a TOF extraction frequency of 83.3 kHz and was operated in continuous mode to collect mass spectra at 1000 kHz. A full mass spectrum (*m*/*z* 7–280) was measured systematically with each datapoint.^57^ The instrument was equipped with a notch filter, which allowed to selectively attenuate intense signals from abundant species such as plasma-gas ions (N_2_^+^, H_2_O^+^, O_2_^+^, Ar^+^) as well as matrix ions (Na^+^) thereby protecting the detector.^57^ Cell suspensions were introduced using a single-cell sample introduction system (SC-SIS, Glass Expansion Inc., Australia) in combination with a manual syringe (KD Scientific) at a flow rate of 10 μl min^−1^.^58^ This dedicated sample introduction system consisted of a low flow pneumatic nebulizer (MicroMist HE U-Series Nebulizer 0.2 mL min^−1^) and a total consumption spray chamber, in which the use of a sheath prevented sample deposition and improved transport efficiency. The nebulization efficiency was assessed using a dilute suspension of commercially available monodisperse spherical 50 nm Au nanoparticles (50.1 nm ± 1.8 nm, original PNC 3.9 × 10^10^ particles per mL, NanoComposix, San Diego, CA, USA). By applying the particle frequency method,^59^ the nebulization efficiency of the system was determined to be 83 ± 3%. The instrument was operated in collision cell mode with 5 mL min^−1^ of a premix (He + 7% H_2_) to remove the interference of ArO^+^ on ^56^Fe^+^. A detailed list of operating conditions can be found in Table S1 in the ESI.† For each sample, >1000 cells were measured.

**Fig. 1:**
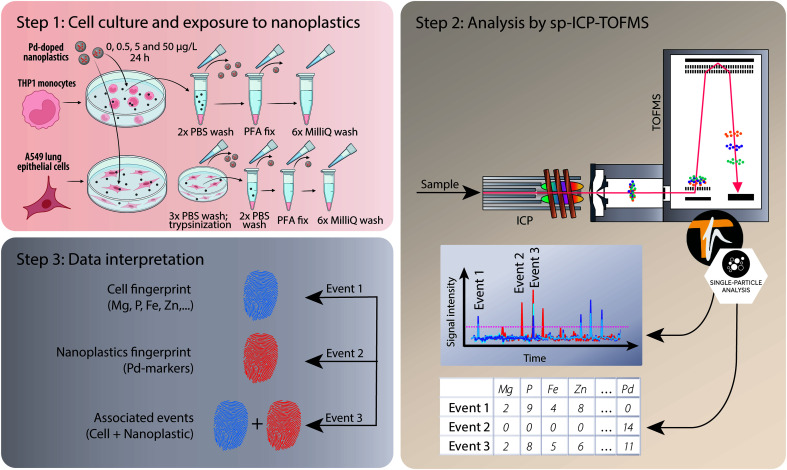
Overview of the experimental design. After cell culture, exposure to model Pd-doped nanoplastics, and washing (step 1), the different cell samples were introduced into the ICP *via* a dedicated single-cell sample introduction system (step 2). Using the ICP-TOFMS, which provides high-sensitivity full-spectrum mass analysis at high time resolution, multi-elemental fingerprints of each particle event were measured, and these could then be assigned to different sub-populations, *i.e.* cells, nanoplastics or associated events (cells + nanoplastics) during data processing (step 3). Portions of the figure were created with https://Biorender.com.

After acquisition, the data were then processed in TOFpilot (v2.10, TOFWERK AG, Thun, Switzerland) using the *LiquidReprocessing* module, which included data thresholding, *i.e.* identification of discrete events such as cells, nanoplastics or associated events from the baseline, followed by split event correction and baseline subtraction. These steps allowed us to refine the dataset to the selected analytes of interest. Integrated signal intensities for the selected nuclides (^24^Mg, ^31^P, ^56^Fe, ^64^Zn and ^65^Cu targeting cell events as well as the Pd isotopes ^104^Pd, ^105^Pd, ^106^Pd, ^108^Pd and ^105^Pd targeting nanoplastics events) were then exported in CSV format for further data analysis, using R-scripts (R Statistical Software, v4.1.2; R Core Team 2021) written in-house. An overview of the experimental design from cell culture to analysis and data interpretation is presented in Fig. 1.

### 2.4 Cell sample preparation for transmission electron microscopy (TEM)

Cells were seeded (3.33 × 10^6^ THP-1 cells in 6 mL CM or 2.67 × 10^6^ A549 cells in 5 mL CM per T25 flask) and cultivated for 4 h before addition of nanoplastics (0 or 50 μg L^−1^). After 24 h of exposure, THP-1 cells were directly collected, and the cell pellet was washed twice with PBS. The adherent A549 cells were first washed once with PBS, trypsinized and the cell pellet washed twice with PBS. Cells were fixed with 2.5% Glutaraldehyde (Sigma-Aldrich) in 0.1 M Na-cacodylate buffer (Electron Microscopy Sciences) for 2 h, washed twice with 0.1 M Na-cacodylate buffer and stored at 4 °C in fresh 0.1 M Na-cacodylate buffer for several days. Samples were stained with 1% osmium tetroxide (Electron Microscopy Sciences) in 0.1 M Na-cacodylate buffer for 1 h in the dark by incubation at room temperature. After three washes with MilliQ water, the cell pellets were dehydrated using an ethanol series (30%, 50%, 70%, 90%, 100%), incubated in a mixture of 1 : 1 ethanol 100% and Epon 812 substitute (Epoxy embedding kit 45359, Sigma-Aldrich) for 1 h and kept overnight in 100% Epon. The samples were subsequently embedded in fresh Epon inside molds and cured for 4 days in an oven at 60 °C. Ultra-thin sectioning from the resin blocks was performed using an ultramicrotome (Leica EM UC6) with an ultra 35° diamond knife (Di-ATOME). The 70–100 nm thick sections were placed on Formvar-coated copper grids (200 mesh, EM resolutions) and were imaged by transmission electron microscopy with a Zeiss EM 900 microscope (Carl Zeiss Microscopy GmbH, Germany) at 80 kV and different magnifications.

## 3. Results and discussion

### 3.1 Nanoplastics characterization by sp-ICP-TOFMS

**Fig. 2:**
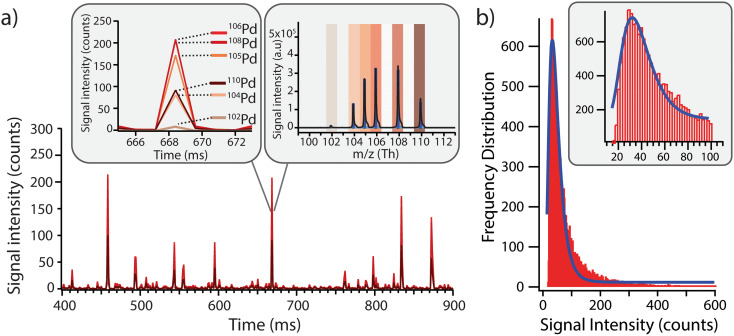
a) Model Pd-doped nanoplastics were diluted in MilliQ, where spikes above the baseline correspond to individual nanoplastics through sp-ICP-TOFMS analysis. Insert: Pd isotopic abundances in the nanoplastics were in line with naturally occurring Pd abundances, where Pd was distributed over six stable isotopes: ^102^Pd (1.02%), ^104^Pd (11.14%), ^105^Pd (22.33%), ^106^Pd (27.33%), ^108^Pd (26.46%) and ^110^Pd (11.72%). Because ^106^Pd and ^108^Pd are the two most abundant isotopes, these were used as markers for identifying Pd-nanoplastics in subsequent experiments. b) ^106^Pd-signal intensity frequency distribution of the Pd loading across the nanoplastics population. A log normal distribution was fitted to the data yielding an average value of 32 ± 7 counts for each nanoplastics event.

Before exposing the cells to the model Pd-doped nanoplastics, it was important to first assess the feasibility of detection of the nanoplastics in single-particle mode and to streamline the data processing for nanoplastics-only events. Using a dilute suspension of nanoplastics (approx. 1 × 10^5^ particles per mL), the individual nanoplastics were recognized as spikes above the baseline, corresponding to number of nanoplastics in suspension (Fig. 2a). All Pd isotopes were detected in one particle with signal intensities proportional to natural Pd isotope abundances (Fig. 2a, insert). Because ^106^Pd and ^108^Pd are the two most abundant isotopes, these were used as markers for the identification of the nanoplastics for the rest of the analysis. Different pulse intensities were observed for each nanoplastics particle, which was likely due to a slightly different Pd loading in each particle and/or some particle agglomeration during dilution. From the pulse intensities measured across >18 000 nanoplastics events, with intensities ranging from 10 to >500 counts, a signal intensity histogram was built (Fig. 2b) to assess the average Pd loading per particle. Here, a log-normal fit was applied to the histogram yielding an average signal intensity of 32 ± 7 counts. Although the fit did not exhibit an ideal match with the data, indicating some deviations from the log-normal distribution, the majority of the nanoplastics presented a central signal intensity around 32 counts. Consequently, the Pd signal intensity per particle was sufficient to detect these model nanoplastic and it should be possible to assess the number of nanoplastics associated per cell by using the average signal intensity per particle and dividing the total intensity associated with each cell.

### 3.2 Method development and performance of sample introduction system for single-cell analysis

The sample introduction system for single cells was fine tuned to ensure cell integrity (*i.e.*, reduce likelihood of cell rupture) during transport into the plasma. Due to the backpressure at the tip of the nebulizer, a high nebulizer flow rate would result in cell rupture and lead to an increased detection of cell fragments as well as an elevated ionic background of cell elements. Therefore, the gas flows needed to be optimized and the optimization criteria was set on event counts as opposed to signal intensity (Table S1†). Initially the syringe pump was placed horizontally but the cell count gradually decreased between successive measurements replicates, indicating cells settling over time, which did not allow for the measurement of a homogeneous suspension. However, when the syringe pump was rotated vertically, the force of gravity prevented cell settling and a consistent number of cells were observed between replicate measurements (Fig. S2†).

Because the two cell types under investigation in this study are significantly larger than the 100 nm Au NPs, the nebulization efficiency was re-evaluated based on the initial cell concentrations. Nebulization efficiency values ranged between 14% and 33% for THP-1 cells and between 2% and 6% for A549 cells. Previous literature reported higher nebulization efficiencies for the sample introduction system used here, namely TE > 70% for bacteria. However, it should be noted that human cells are typically larger (10–20 μm in diameter) than bacterial cells (1–5 μm in diameter) and a reduced nebulization efficiency is in line with findings from previously published work showing an inverse correlation between cell size and nebulization efficiency.^60^ While these findings suggest that similar size standards should be used for an accurate assessment of the transport nebulization in future studies, a well-characterized μm-sized standard for cell analysis is still lacking to the best of the authors' knowledge. Furthermore, following the increased interest in single-cell ICP-MS applications, different dedicated systems to further improve the nebulization efficiency of cells have been developed. For example, Menero-Valdés *et al.* used a commercially available microFAST Single Cell set-up and reported a nebulization efficiency of 81% for platinum nanoparticles and of 51% for ARPE-19 cells.^61^ In a different study, the robustness and reproducibility of this autosampler was successfully demonstrated for nanoparticles analysis and holds great promises for high-throughput single-cell analysis.^62^

### 3.3 Detection of THP-1 and A549 cells by sc-ICP-TOFMS based on their elemental fingerprint

In contrast to mass cytometry, where cells are identified based on heavy metal-conjugated probes or antibodies in order to recognize cell-type specific intracellular or extracellular targets or antigens, here the cells are detected based on their elemental composition. The ICP-TOFMS measures the full mass spectrum for each cell without any prior knowledge of the elements present, thereby allowing the determination of the endogenous elemental fingerprint of the cells.^63^ For the cells analysed here, magnesium, phosphorous, iron, copper and zinc presented detectable signals and were therefore identified as potential cellular fingerprint elements. Magnesium is an essential mineral involved in DNA and RNA synthesis, as well energy metabolism and ion transport. Phosphorus is a fundamental component of nucleic acids, phospholipids, and energy-carrying molecules (*e.g.* ATP), making it an essential component of living cells. Iron is key component of heme in hemoglobin (oxygen transport) and is involved in redox reactions within the cell. Copper serves as a cofactor for enzymes involved in cellular metabolism. Zinc participates in various cellular processes, such as DNA repair, gene expression, and immune function. Although the elements listed above could be expected to be present in cells, their detection was not consistent due to variability in cell size and respective elemental content, as well as element-specific instrumental sensitivity and limits of detection. For example, while magnesium and iron were observed in many cells, the high variability of magnesium content in some samples hindered its use as a reliable cell marker. Additionally, iron detection was limited in smaller cells due to insufficient sensitivity, leading to inconsistent results. Consequently, using all five elements as ‘must-haves’ for defining a cell event would result in a reduced number of total cell events, as some cells might have signals below the detection threshold for certain elements, leading to false negatives. To address this, we refined the cell selection criteria during data analysis and concluded that a combination of phosphorous (^31^P) and zinc (^64^Zn) signals, recorded simultaneously, provided the most reliable indication of a cell event independent of cell size. This approach reduced the risk of false negatives and facilitated the accurate assignment of cell event to recorded events.

### 3.4 Single-cell analysis of nanoplastics uptake in and association with human cells

As opposed to mass cytometry, the method developed here focusses on direct, label-free analysis of single cells by ICP-TOFMS while simultaneously being able to detect the Pd incorporated into the nanoplastics. Indeed, while palladium-based labelling reagents are available to stain cells,^64^ here the cells were detected based on their elemental fingerprint and therefore the use of Pd-doped nanoplastics did not impinge upon cell detection. Consequently, the use of Pd-tagged nanoplastics combined with sc-ICP-TOFMS provided an elegant approach to quantify nanoplastics association with cells along with the parallel detection of multi-elemental fingerprints of each cell in a label-free manner.

**Fig. 3:**
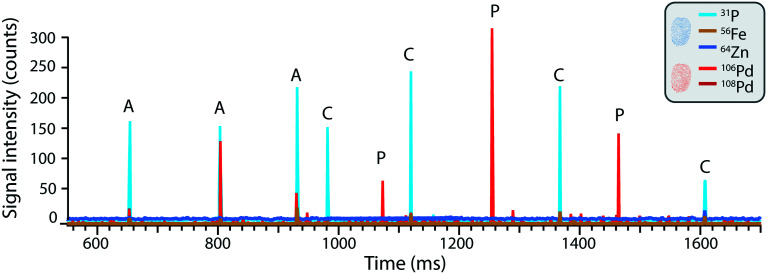
The time trace of THP-1 cells exposed to 5 μg L^−1^ Pd-doped nanoplastics where different species can be recognized as pulses above the background. Namely: (C) single THP-1 cells without associated nanoplastics, (P) nanoplastics and (A) associated events.

The simultaneous identification of different analytes of interest allowed for multiplexed sc-ICP-TOFMS experiments. As shown in Fig. 3, cells were recognized based on the concurrent detection of ^31^P and ^64^Zn and nanoplastics on the concurrent detection of ^106^Pd and ^108^Pd, respectively. Subsequently, signals which simultaneously presented the cellular fingerprint (^31^P and ^64^Zn) and the nanoplastics fingerprint (^106^Pd and ^108^Pd) were considered associated events. This simultaneous multi-element detection is particularly advantageous in this case as it allowed us to have a direct assessment of nanoplastics–cell association. Even so, while association is easily determined, it is not possible to distinguish between internalized and cell surface-associated particles. While concurrent signals typically represent nanoplastics–cell association, there is some probability that a cell and a nanoplastic enter the plasma simultaneously. The probability of such cases was calculated using Poisson-based concurrence analysis (see ESI† for additional explanation) and such instances accounted for less than 1% of all cases where cells and nanoplastics were measured concurrently. By further diluting the suspension, the probability of such false positive associations would be further reduced.

In order to assess the dose-dependent uptake through sc-ICP-TOFMS, we exposed THP-1 and A549 cells to three different nanoplastics concentrations (0.5 μg L^−1^, 5 μg L^−1^, and 50 μg L^−1^) and a control (0 μg L^−1^) for 24 hours. Although the concentrations were not intended to represent realistic concentrations, but rather to assess the feasibility of the method, a recent study detected average microplastic concentrations of 1.6 μg mL^−1^ in human blood,^8^ which is above our highest exposure concentration of 0.05 μg mL^−1^. Lower concentrations of nanoplastics could be measured, but analysis time would need to be increased in order to capture enough events for a robust statistical analysis. It is important to note that the study serves as a proof-of-principle for this methodology and that by using elevated exposure concentrations, we would increase the number of nanoplastics/cell interactions. Consequently, this facilitated assessment of the method, but we would envision that one would also be able to measure lower concentrations of nanoplastics (and their association with cells) should exposure concentrations be reduced. Furthermore, the strength of the method relies on its adaptability and its potential for automation through use of an autosampler allowing for unattended operation^62^ and automated data processing, which together allow for the analysis of more cells and would facilitate capturing more infrequent nanoplastics/cells associations at reduced exposure concentrations.

**Fig. 4:**
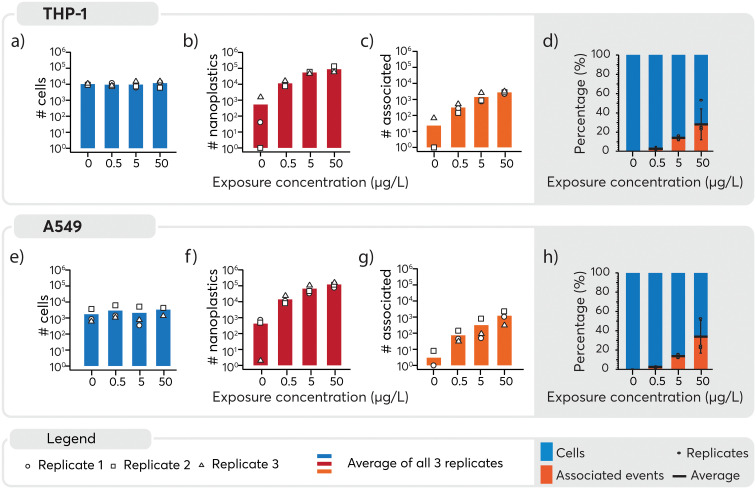
Overview of the number of cells events (panels a and e), nanoplastics events (panels b and f) and associated events (panels c and g) recorded per cell type and per exposure concentration for all replicates. Solid bars indicate averages across experimental exposure replicates and the average of each replicate is indicated by the open symbol. The total number of cells and nanoplastics measured in each replicate varied, but typically at least 1000 cell events were measured per replicate. The average association of cell–nanoplastics after normalization to the total number of cells per replicate is shown in panels d) and h). The average and individual replicates are represented by a horizontal black line and black dots, respectively. The standard deviation was calculated on the normalized data and added to the bar plot. Overall, as the nanoplastics exposure is increased, a dose-dependent increase in particle association is observed for both cell lines.

An overview of the number of cells, nanoplastics and associated events detected under the different exposure conditions is presented in Fig. 4. A similar number of cells were detected across all samples (Fig. 4, panels a and e) and, as expected, increasing numbers of nanoplastics were observed with higher exposure doses (Fig. 4, panels b and f). Consequently, nanoplastics–cell association was positively correlated with the exposure concentration for both cell types (Fig. 4, panels c and g). Some nanoplastics were detected for non-exposed cells, likely originating from cross-contamination during the analysis. Despite this, the total number of particles was an order of magnitude lower for 0 μg L^−1^ than 0.5 μg L^−1^ samples, where only eight associated events were detected across approximately 4000 cell events measured for the unexposed cell population. Consequently, the impact of the cross-contamination was considered to be negligible for the results overall. The observed concentration-dependent association of nanoplastics is in line with similar data for other nanoparticles.^65,66^ Subsequently, to better understand cell-type specific differences in cellular association of nanoplastics, data were normalized to the total number of cells (Fig. 4, panels d and h). For THP-1 cells, the percentage of associated particles was 0% in the control group, 3% at 0.5 μg L^−1^, 14% at 5 μg L^−1^, and 28% at 50 μg L^−1^ nanoplastics exposure (see Table S2 in ESI†). Similarly, for A549 cells, the percentage of associated particles was 0% in the control group, 3% at 0.5 μg L^−1^, 14% at 5 μg L^−1^, and 33% at 50 μg L^−1^ nanoplastics exposure. These findings demonstrate that both THP-1 and A549 cells exhibited a dose-dependent association with nanoplastics and have a similar endocytotic capability for the nanoplastics measured here.

In the assessment of nanoplastics–cell association, two aspects need to be considered: 1) the proportion of cells that are associated with nanoplastics, 2) the number of nanoplastics associated with each cell and 3) the heterogeneity or homogeneity observed in the association pattern. It should be noted that as cells also contain carbon, distinguishing between the carbon content in cells and the carbon in nanoplastics adds another level of complexity. While it is possible to distinguish μm-sized plastics from algae cells using multi-element fingerprints,^22^ the low intrinsic carbon mass of nanoplastics and limited sensitivity for carbon, prevented their detection and thus reinforces the need to explore alternative approaches such as using metal signals as proxies for the nanoplastics. Consequently, here Pd-signals were used as proxy for individual nanoplastics detection. Subsequently, based on the average signal intensity measured for each nanoplastics (Fig. 2b), the magnitude of the nanoplastics spike intensity associated with each cell served as a surrogate to determine the approximate number of nanoplastics associated with each cell (Table S3†). Consistent with previous results, a higher number of nanoplastics were associated with higher exposure doses. However, large standard deviations were observed when calculating the average number of associated nanoplastics, indicating a rather heterogeneous association. A closer look into the data revealed that in numerous cases, a very high signal intensity was observed for Pd which could be explained by multiple nanoplastics being associated with the one cell or the association of a large agglomerate with one cell. Because these larger values can act as outliers and significantly impact the mean and standard deviation, the median, which is less sensitive to extreme values, was determined. It provided a more robust estimate of the main tendency and showed that an increase in the number of nanoplastics associated was observed with increasing concentration. A larger number of nanoplastics were found to be associated at 50 μg L^−1^ exposure as opposed to 0.5 and 5 μg L^−1^. Overall, a rather heterogeneous association between cells and nanoplastics was observed. Furthermore, a substantial proportion of cells did not exhibit any association with nanoplastics. This finding underscores the diversity of interactions between cells and nanoplastics, highlighting the need for further investigation into the factors influencing these associations.

**Fig. 5:**
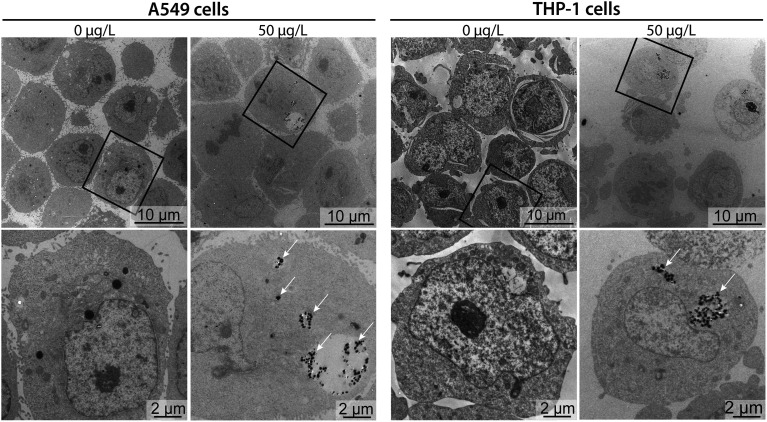
Cellular uptake of nanoplastics. TEM micrographs of A549 and THP-1 cells exposed to 0 μg L^−1^ (untreated controls) or 50 μg L^−1^ of nanoplastics for 24 h. Lower row images are higher magnifications of the cells marked in the upper row (rectangle). Clusters of internalized nanoplastics are indicated with white arrows.

To better contextualize the associations measured by sc-ICP-TOFMS, and to approximate whether these measurements corresponded to nanoplastics cell internalization or surface adhesion, transmission electron microscopy (TEM) images were acquired for cells exposed to 50 μg L^−1^ nanoplastics. Micrographs confirmed that nanoplastics were indeed internalized by both cell types (Fig. 5). Clusters of particles were observed mostly in endocytic vesicles present in the cytosol, indicating the involvement of active endocytotic uptake mechanisms. This finding is similar to a previous study, showing the involvement of several endocytotic uptake mechanisms (*e.g.*, phagocytosis, micropinocytosis, clathrin- and caveolin-independent endocytosis) in the uptake of 40 nm polystyrene nanoparticles in J774A.1 mouse macrophages and A549 epithelial cells in dependence of particle size and cell type.^18^ Moreover, particles were only found in some of the cells, which is in line with the sc-ICP-TOFMS data, where association was observed in approximately 30% of the cells. Furthermore, from the images it can be seen that the cells contained different numbers of nanoplastics per cell, again highlighting a heterogeneous association, supporting sc-ICP-TOFMS findings shown in Table S3.† However, TEM is not well suited for quantitative analysis and some cells might contain particles in areas outside of the imaged section. Indeed, here, the selected images show a rather larger clustering of nanoplastics, which would support the observed large mean and standard deviation presented in Table S3.† However, it is possible that in another section fewer nanoplastics would be observed. Consequently, by combining these techniques, we have achieved a more complete picture of nanoplastics–cell interactions, leveraging the strengths of each analytical approach. The high-throughput capability of sc-ICP-TOFMS provided a robust statistical analysis of nanoplastics associations across a large-scale experiment, while TEM offered detailed insights into the internalization of nanoplastics at the cellular level.

## 4. Conclusions

Collectively, sc-ICP-TOFMS holds significant potential for assessing particle association in biological systems. First, it offers faster throughput compared to techniques such as transmission electron microscopy (TEM) and energy-dispersive X-ray spectroscopy (EDX) for assessing particle association. This accelerated analysis allows for high-resolution measurements of individual cells in a shorter time frame. Second, by measuring the full mass spectrum, sc-ICP-TOFMS enables the detection of single cells based on their intrinsic elements, eliminating the need for labeling and reducing potential biases or artifacts in the analysis. Third, measuring particle association on a cell-by-cell basis enables a more accurate assessment of biological variability. In this way, sc-ICP-TOFMS allows researchers to capture the heterogeneity within a sample and better understand the distribution and localization of particles within and amongst different cell populations. Finally, sc-ICP-TOFMS has the potential to provide elemental quantification at extremely low levels, allowing for the detection and analysis of trace elements, nanoparticles, or in this case metal-labeled nanoplastics that may be missed by other techniques. This capability opens up new possibilities for studying subtle interactions between cells and particles. Further research is needed to optimize and standardize this technique for widespread use, and improvements could include: 1) high(er) sample throughput, with possible automation and higher transport efficiency specifically for cells and 2) a more streamlined data post-processing workflow and normalization procedures to accommodate the large information density gained in such a multiparametric analysis. Nevertheless, the advantages outlined above position sc-ICP-TOFMS as a promising tool for advancing our understanding of particle–cell interactions and their implications in various biological systems.

Here, the sc-ICP-TOFMS method allowed us to successfully detect model nanoplastics with a Pd-label as well as their association with human cells. We observed a dose-dependent uptake of nanoplastics in THP-1 monocytes and A549 alveolar epithelial cells and quantification data at single cell level revealed similar numbers of associated nanoplastics with both cell types. Interestingly, uptake was highly heterogeneous within the same cell population, with some cells being associated with many particles while others remained nanoplastics free. This has important implications for future hazard assessments of nanoplastics, since cells with higher particle number association, especially uptake, may exhibit differential bioresponses compared to cells without associated particles. While the current study focused on the effect of one exposure time, namely 24 h, the time-dependent aspect should not be neglected and future studies should investigate different exposure times to also understand time-dependent associations of nanoplastics. Therefore, advanced single-cell techniques such as single-cell RNAseq could be used to complement the single cell particle association data obtained in this study to decipher toxicity responses of nanoplastics. Although this study was performed with relatively simple *in vitro* monocultures, the sc-ICP-TOFMS approach developed here could be explored for advanced co-culture models, organoid cultures or *ex vivo*/*in vivo* tissues.

Overall, assessment of human health hazards of nanoplastics is currently a high priority research area, however, researchers are facing major challenges in the detection of nanoplastics in biological tissues. sc-ICP-TOFMS could provide a promising analytical technique for the sensitive and fast quantification of metal-labeled nanoplastics at single cell level in order to complement other approaches and gain novel insights into potential toxicity mechanisms. Furthermore, while the current study focuses on metal-doped nanoplastics, real-life plastic debris contain metal additives, which could potentially be used as tracers for the nanoplastics.

## Conflicts of interest

There are no conflicts to declare.

## Supplementary Material

EN-010-D3EN00681F-s001
