# Supplementary material for: Development of single-cell ICP-TOFMS to measure nanoplastics association with human cells
Source: Environ Sci Nano. 2023 Nov 9;10(12):3439–49. doi: 10.1039/d3en00681f (PMC10702630; doi:10.1039/d3en00681f)
Supplement: EN-010-D3EN00681F-s001 [file EN-010-D3EN00681F-s001.pdf]

Electronic Supplementary Material (ESI) for Environmental Science: Nano.
This journal is © The Royal Society of Chemistry 2023

Supporting Information

Development of single-cell ICP-TOFMS to measure nanoplastics association with human cells

Lyndsey Hendriks[1], Vera M. Kissling[2], Tina Buerki-Turnherr[2], Denise M. Mitrano[3]

1. TOFWERK AG, Schorenstrasse 39, 3645 Thun, Switzerland
2. Laboratory for Particles Biology Interactions, Swiss Federal Laboratories for Materials Science and Technology (Empa), Lerchenfeldstrasse 5, CH-9014, St Gallen, Switzerland
3. Department of Environmental Systems Science, ETH Zurich, Universitätstrasse 16, 8092 Zurich, Switzerland

Initial measurements using sp-ICP-TOFMS revealed a notable presence of sodium and phosphorus in the cell media, resulting in a high ionic background in the cells samples. Additionally, a high Pd-background was measured resulting from nanoplastics which were not associated with cells. These high background signals masked both single cell events (spikes) in the dataset and made the assessment of determining nanoplastics-cell association more challenging. Together, this necessitated the optimization of the sample preparation process. While sodium is typically regarded as a marker for cell viability, its high background signal prevented the detection of individual spikes produced by cells, thus its signal was actively suppressed using the notch filter. On the other hand, the background phosphorus signal was several orders of magnitude lower than that of sodium, and because some signals could be identified above the background, phosphorous was consequently identified as a significant cell marker. To reduce the background phosphorus signal to more clearly distinguish cell events, additional washing steps or cell suspension dilution was carried out, depending on the cell type. These washing steps also facilitated the removal of unbound nanoplastics from the suspension before sc-ICP-TOF-MS analysis. A549 lung epithelial cells are adherent cells that can be effectively washed while remaining attached to the cell culture plastic surface, facilitating the removal of unbound particles. In contrast, THP-1 monocytes are free-floating cells in suspension. Washing suspension cells involves centrifugation and re-dispersion steps, which are more susceptible to cell losses, cell breakage and co-precipitation of unbound particles. The original washing procedure did not completely eliminate all unbound nanoplastics. Thus, when measuring the highest nanoplastics exposure concentration (50 µg/L), a substantial number of unbound nanoplastics were observed in the pellet, leading to an artificially high Pd background that hindered the identification of individual nanoparticle events. Consequently, further optimization of the washing procedure was required to achieve two goals: 1) reduction of the ionic background from the cell media and 2) removal of unbound nanoplastics. To evaluate the effectiveness of the washing steps and to determine the optimal number of washes, the detection of nanoplastics as well as the level of the background elements was assessed by systematically measuring the supernatant of the THP1-cells, which were the most challenging to wash. The results, presented in Figure S1, demonstrated a significant reduction in both the ionic background and the number of unbound nanoplastics with the implementation of four additional washing steps.

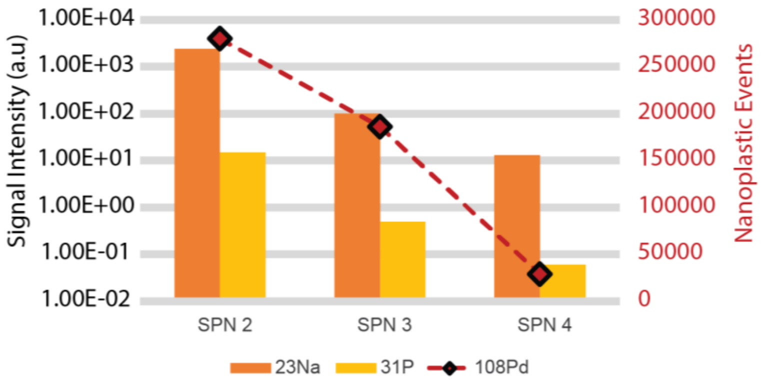

Figure S1 – Through analysis of the supernatant analysis, the washing procedure was optimized to decrease the ionic background and to remove unbound nanoplastics from the exposure media. The sample preparation optimization was conducted on the THP-1 cells as these were more challenging to wash compared to the adherent A549 cells. SPN2, SPN3 and SNP4 refers to the supernatant after washes 2, 3 and 4, respectively.

Table S1: Instrument operating conditions for sc-ICP-TOFMS

Parameter	Value
Injector	1.5 mm sapphire
Sample flow rate [µL min^{-1}]	10
Nebulizer gas [L min^{-1}]	0.32
Add. Gas [L min^{-1}]	0.9
CCT Gas (He + 7% H_2) [L min^{-1}]	5
Sampling depth [mm]	3
Plasma power [W]	1550
Auxiliary flow [L min^{-1}]	0.8
Cool Flow [L min^{-1}]	14

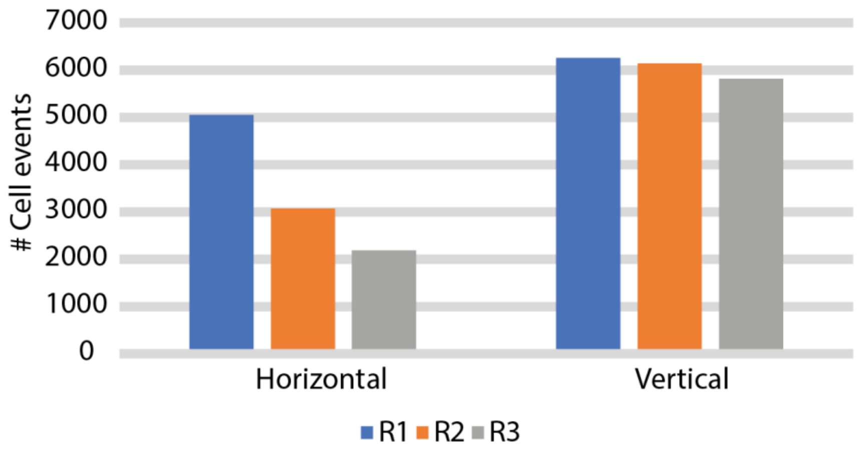

Figure S2 – Optimization of the sample introduction setup with the syringe pump applied to the THP1 cells. Initially, with the horizontal arrangement, a pronounced decrease in cell counts was observed during successive measurements performed over an 8-minute analysis interval. Specifically, between the first and second measurements, the THP1-cell count exhibited a significant drop of 40%, followed by a subsequent decrease of 30%. In contrast, by setting the sample introduction system in a vertical position, a considerably more stable THP1-cell count was recorded over time. Over the course of three consecutive runs, the THP1-cell count showed only minor decreases of 2% and 5% cell number counts, respectively. This approach allowed us to more accurately and consistently measure cells from a homogeneous suspension.

Concurrent event analysis

It is important to note that although concurrent signals are assumed to represent an association between nanoplastics and cells, there is a possible scenario in which these signals might originate from an independent nanoplastic and an independent cell entering the plasma at the same time. In this case, the nanoplastic and the cell will be measured concurrently and considered as associated when, in reality, they are not. Retrospectively determining which scenario occurred is not possible, but the likelihood of such cases can be calculated using simple concurrency analysis.[a] The probability of both independent signals being detected simultaneously ($P_{Cells \cap Plastics}$) can be calculated as the product of the individual probabilities of measuring a single cell (P_{Cells}) and a nanoplastic ($P_{PLastics}$) at a given time point:

$$P_{Cells \cap Plastics} = P_{Cells} \times P_{Plastics}$$

Where the individual species probabilities P_x (x = cell or nanoplastic) is given by [b]

$$P_x = \frac{\lambda^x}{x!} \cdot e^{-\lambda}$$

Where x is the number of events and λ is the number of events measured per second entering the plasma divided by the time required to measure the signal from one single event. Consequently, we computed the probability of cell and nanoplastic signals being detected coincidently for the different cell types and exposure concentrations, which resulted in less than 1% concurrent events. Therefore, while there remains a possibility that our assumption of nanoplastic association with cells is a false positive, the probability of this scenario remains low, even for the highest exposure concentration.

[a] Mehrabi K, et al, *Environ. Sci.: Nano*, **2019**, 6, 3349
[b] Olesik J, et al, *J. Anal. At. Spectrom.*, **2012**, 27, 1143

Table S2: Overview of the normalized cellular association data for nanoplastics by cell type and exposure concentration used in Figure 4.

		THP1				
		R1	R2	R3	Average	STD
Exposure Concentration (µg/L)	0	0.0%	0.0%	0.6%	0.2%	0.4%
	0.5	2.3%	1.9%	6.3%	3.5%	2.4%
	5	12.1%	11.7%	17.5%	13.8%	3.2%
	50	16.5%	46.1%	21.0%	27.9%	15.9%
		**A549**				
		R1	R2	R3	Average	STD
Exposure Concentration (µg/L)	0	0.1%	0.2%	0.0%	0.1%	0.1%
	0.5	3.2%	2.3%	2.9%	2.8%	0.4%
	5	14.1%	15.7%	12.2%	14.0%	1.8%
	50	24.6%	53.1%	22.9%	33.5%	17.0%

Table S3: Estimated number of nanoplastics associated per cell depending on cell type and exposure concentration, as measured by sc-ICP-TOFMS, taking into account the average Pd mass loading per nanoplastic. As there is a large standard deviation, the association appears to be very heterogeneous across the cells measured. While considering the effect of heterogeneous uptake on the mean and standard deviation, the use of the median helped mitigate the impact of extreme values and provided a more reliable and interpretable measure of association. As with the percentage of association, a similar pattern can be observed, with an increasing number of associated nanoplastics with increasing exposure concentration which is similar for both cell types.

**Average ± Stdev**		THP1			A549		
		R1	R2	R3	R1	R2	R3
Exposure Concentration (µg/L)	0.5	1.1 ± 1.7	11.7 ± 23.3	1.4 ± 3.1	3.6 ± 9.8	7.3 ± 13.7	1.4 ± 1.1
	5	2.4 ± 4.9	16.6 ± 38.1	2.5 ± 5.5	7.0 ± 12.3	14.5 ± 34.0	2.1 ± 2.3
	50	8.8 ± 13.4	37.2 ± 57.7	16.2 ± 29.5	21.8 ± 35.3	29.4 ± 49.8	11.8 ± 31.5

**Median**		THP1			A549		
		R1	R2	R3	R1	R2	R3
Exposure Concentration (µg/L)	0.5	1.0	1.3	0.7	1.0	1.0	1.0
	5	2.2	1.6	1.2	1.9	1.6	1.2
	50	4.9	11.5	8.4	11	8.9	3.5
